# Machine learning innovations for reliable gurney energy estimation in energetic materials

**DOI:** 10.1016/j.isci.2026.116536

**Published:** 2026-06-25

**Authors:** Mingyue Deng, Raouf Hassan, Alireza Baghban

**Affiliations:** 1 School of Safety Engineering and Emergency Management, Nantong Institute of Technology, Nantong, Jiangsu 226002, China; 2 Department of Computer Engineering, Mokwon University, Daejeon 35349, South Korea; 3 Civil Engineering Department, College of Engineering, Imam Mohammad Ibn Saud Islamic University (IMSIU), Riyadh 13318, Saudi Arabia; 4 Department of Process Engineering, National Iranian South Oilfields Company (NISOC), Ahvaz, Iran

**Keywords:** Machine learning, Materials science

## Abstract

Accurately predicting the Gurney energy (E_G_) of energetic materials is crucial for industrial applications, yet it traditionally requires costly, time-intensive empirical testing and complex thermochemical modeling. This study aims to overcome these limitations by developing a robust, high-throughput machine learning framework to predict E_G_directly from intrinsic molecular and thermodynamic descriptors. Following rigorous data standardization, multiple machine learning algorithms were optimized and evaluated to capture complex structure-property relationships. Quantitative analysis revealed that the artificial neural network (ANN) significantly outperformed baseline models, yielding exceptional predictive accuracy (R^2^ = 0.995 and MRD <1.5%). Feature importance analysis demonstrated that produced nitrogen gas (N_2_), oxygen balance, and solid carbon yield (C) are the most dominant predictors, strongly aligning with fundamental detonation physics. Ultimately, this data-driven approach provides a highly accurate and efficient alternative to traditional measurements, offering profound practical significance for the rapid in silico screening and targeted formulation design of energetic materials.

## Highlights

• Developed predictive models for Gurney energy using AI techniques
• Achieved high accuracy with ANN models, R^2^ up to 0.979
• Identified key predictors: nitrogen gas, oxygen balance, solid carbon yield
• Enhanced efficiency in estimating Gurney energy for construction materials

## Graphical abstract

**Figure:**
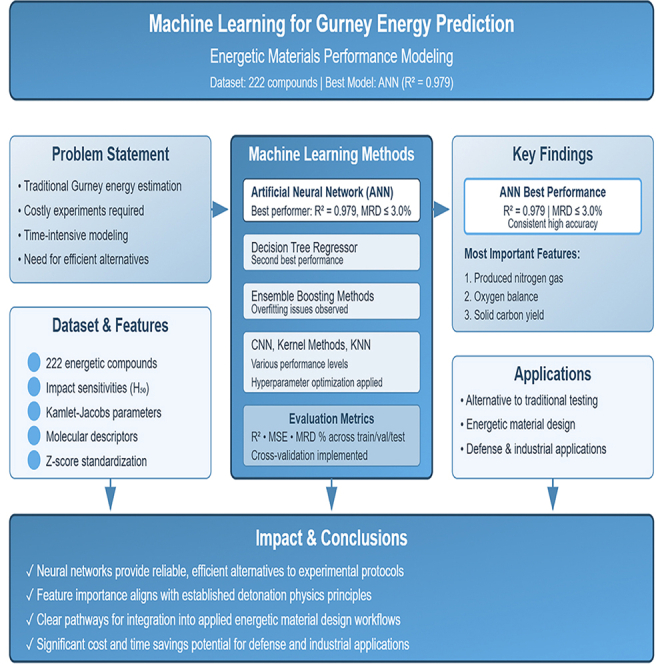

## Introduction

The performance of energetic materials is a subject of enduring interest in defense, mining, aerospace propulsion, and demolition engineering, where precise control over explosive output is critical to mission success, operational safety, and material efficiency.^1^^,^^2^ Among the various performance metrics used to quantify explosive behavior, Gurney energy—the kinetic energy imparted per unit mass of explosive products—serves as a key determinant of metal acceleration, fragment velocities, and overall detonation effectiveness. This parameter encapsulates the ability of an explosive to convert stored chemical energy into mechanical motion, a property that underpins the design of shaped charges, warheads, linear cutting tools, and other systems where energy transfer must be optimized.^3^^,^^4^ Conventional determination of Gurney energy relies on controlled experiments involving the acceleration of metallic plates or casings by detonating charges, followed by velocity measurement via high-speed diagnostics. While these methods yield reliable results, they are inherently expensive, hazardous, time-consuming, and limited in throughput.^5^^,^^6^

The growing demand for rapid, cost-effective predictive capabilities in energetic material research has driven interest in computational approaches that can bypass physical experimentation.^7^^,^^8^ Traditional calculation routes often employ thermochemical models, such as the Kamlet-Jacobs (KJ) equations, which estimate detonation velocity, pressure, and energy based on material density, heat of detonation, and reaction gas properties. Although these models capture key physics, their reliance on idealized reaction assumptions and limited scope of chemical diversity restricts applicability to complex or formulations. Furthermore, empirical models lack the flexibility to fully exploit the wealth of chemical, structural, and functional descriptors now accessible through cheminformatics and computational chemistry tools.^9^^,^^10^

In recent years, data-driven machine learning (ML) models have emerged as a transformative approach to materials prediction, leveraging large datasets to uncover non-linear relationships between molecular structure and performance. Within energetic materials research, ML techniques have been applied to predict impact sensitivity, detonation velocity, and thermodynamic outputs with promising accuracy.^11^^,^^12^ Neural networks, support vector regression (SVR), ensemble tree methods, and regularized linear models have all shown potential in capturing complex interdependencies among compositional, structural, and thermophysical descriptors. By integrating high-dimensional features—oxygen balance (OB), density, heat of explosion, functional group counts, aromaticity, lipophilicity, and more—ML frameworks can describe energetic behavior beyond the reach of purely mechanistic equations.^13^^,^^14^

The literature reflects a gradual expansion from small, specialized datasets toward broader, standardized repositories encompassing hundreds of compounds. Early work by researchers^15^^,^^16^^,^^17^ systematically compiled impact sensitivities and detonation outputs for nitroaromatic and aliphatic explosives, providing a basis for semi-empirical parameterization. Subsequently, the inclusion of computationally derived descriptors from cheminformatics platforms such as RDKit enabled richer statistical modeling. Gu et al.^18^ applied kernel ridge regression to predict detonation parameters of CHNO energetic compounds, demonstrating improvement over linear models for non-linear trends. Similarly, Liu et al.^19^ developed empirical correlations for energetic density and velocity using optimized OB functions, linking stoichiometry to thermodynamic outputs. These efforts, while valuable, typically relied on small training corpora or focused on a narrow chemical class, limiting cross-domain applicability.

Neural network applications have gained traction as computational resources and data availability have expanded. For example, Fayet et al.^20^ employed multilayer perceptrons to predict flash points and sensitivity metrics, integrating diverse molecular descriptors across hundreds of hazardous materials. In parallel, convolutional neural networks (CNNs) have been adapted to chemical graph or descriptor-matrix inputs, allowing spatially correlated features—such as structural fragment distributions—to contribute to learning. Ensemble tree methods such as random forest and gradient boosting have also proven effective, especially when feature importance ranking is desired for physical interpretability. These models can naturally handle heterogeneous feature scales, noisy inputs, and correlated variables, which are common in energetic materials datasets compiled from mixed experimental and calculated sources.^21^^,^^22^^,^^23^

**Figure 1:**
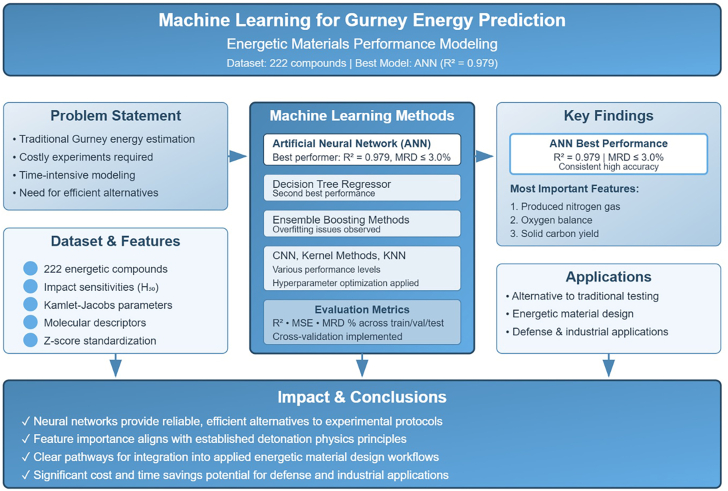
Workflow of the study

Energetic materials play a pivotal role in defense, mining, and propulsion technologies, where precise knowledge of detonation performance directly influences safety, efficiency, and product innovation. In particular, Gurney energy estimation is vital for understanding the kinetic energy distribution in explosive events, yet experimental determination is costly, hazardous, and limited in scalability. This work (See Figure 1 work the workflow) addresses the problem by hypothesizing that computational learning algorithms, trained on a diverse and high-quality dataset, can accurately predict Gurney energy from molecular and physical descriptors. The research workflow comprised dataset curation and preprocessing, descriptor selection through literature-driven cheminformatics analysis, model building with a wide array of algorithms, systematic hyperparameter tuning, and rigorous multi-metric evaluation. The objective was to identify the most reliable, generalizable, and physically interpretable predictive framework, providing a robust computational alternative for Gurney energy estimation.

## Results and discussion

### Hyperparameter optimization

**Figure 2:**
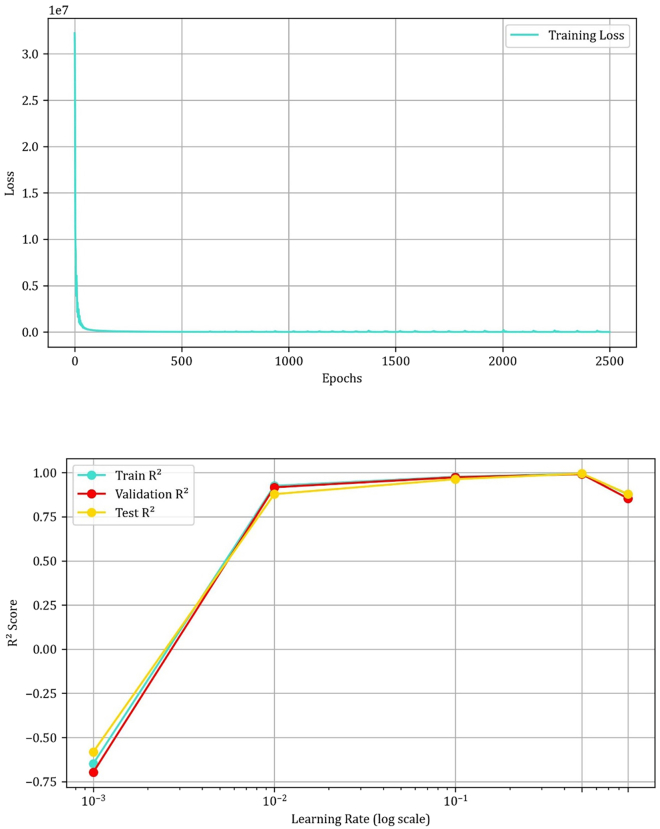
Schematic representation of the artificial neural network (ANN) architecture and learning rate evaluation procedure

Figure 2 shows the ANN training dynamics. The loss curve versus epochs illustrates rapid error reduction within the first ∼400 iterations, followed by a gradual plateau as the network approached convergence at the set maximum of 2500 iterations. This behavior is characteristic of stable weight updates under the chosen ReLU activation in hidden layers and linear output activation, consistent with avoiding vanishing gradients. The R^2^ versus learning rate plot indicates that rates near 10^−3^ achieve the highest predictive accuracy (R^2^ > 0.99), while both lower and higher rates reduce performance due to under-training and unstable oscillations, respectively. The optimized configuration, three layers (32, 8, 1 neurons), balances representation capacity and generalization, avoiding over-parameterization that could cause overfitting.

**Figure 3:**
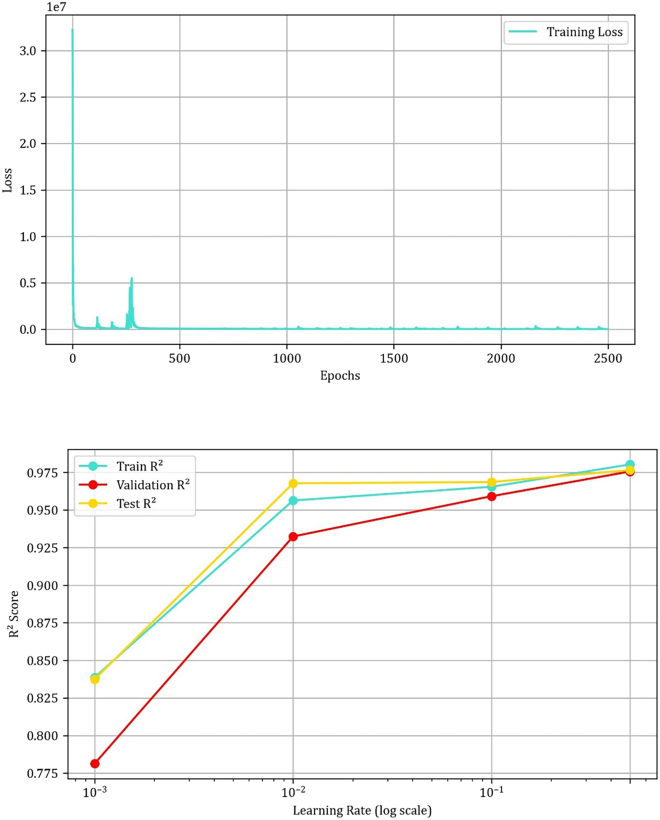
Layout of the convolutional neural network (CNN) architecture and learning rate optimization workflow

For CNN (Figure 3), the loss-epoch curve exhibits a similar early steep descent, with convergence achieved at ∼2000 iterations. The architecture, two convolutional layers (8 and 16 filters) followed by two fully connected layers (32 neurons, 1 output)—successfully reduced dimensionality while retaining spatially correlated features. The R^2^ vs. learning rate plot shows optimal performance at 5 × 10−4, corresponding to the highest model stability and precision in detonation parameter prediction. At extreme learning rates, both underfitting and divergence were observed, highlighting the sensitivity of deep convolutional networks to rate tuning. The ReLU activations in both convolutional and fully connected stages facilitated non-linear feature extraction without degrading convergence speed.

**Figure 4:**
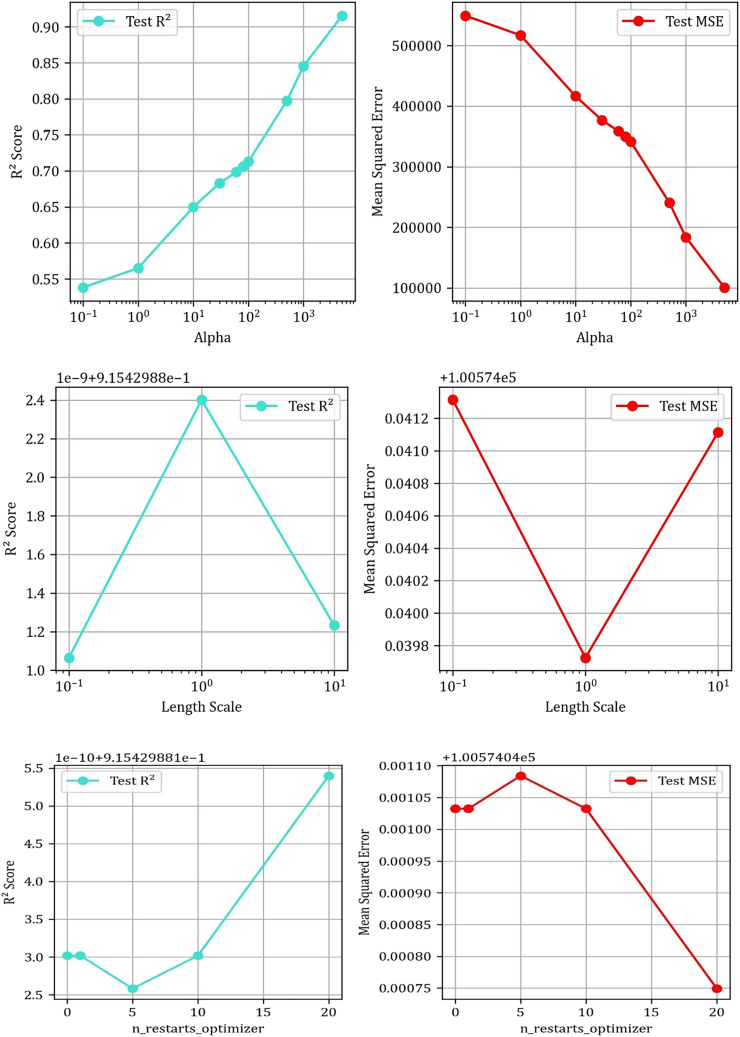
Hyperparameters tuning process for GPR model

Figure 4 indicated tuning process of hyperparameters for GPR model. As can be seen, the best values can be obtained in the minimum mean squared error (MSE) and maximum R^2^ of test data.

Regarding linear regression-based algorithms including simple linear regression, without hyperparameter tuning, provided a baseline R^2^ around 0.91. Ridge and Lasso optimizations demonstrate a strong sensitivity to α∖alphaα, with the optimal value α = 1.6238 yielding R^2^ increases of ∼3–4% relative to poorly tuned cases and achieving MSE reductions of 10%–15%. Elastic net optimization confirmed the best balance between L1 and L2 regularization at α = 0.01274 and I_i_ ratio = 0.2, producing stable R^2^ ≈ 0.94 and the lowest MSE among the regression family indicating effective handling of correlated features without oversparsity.

**Figure 5:**
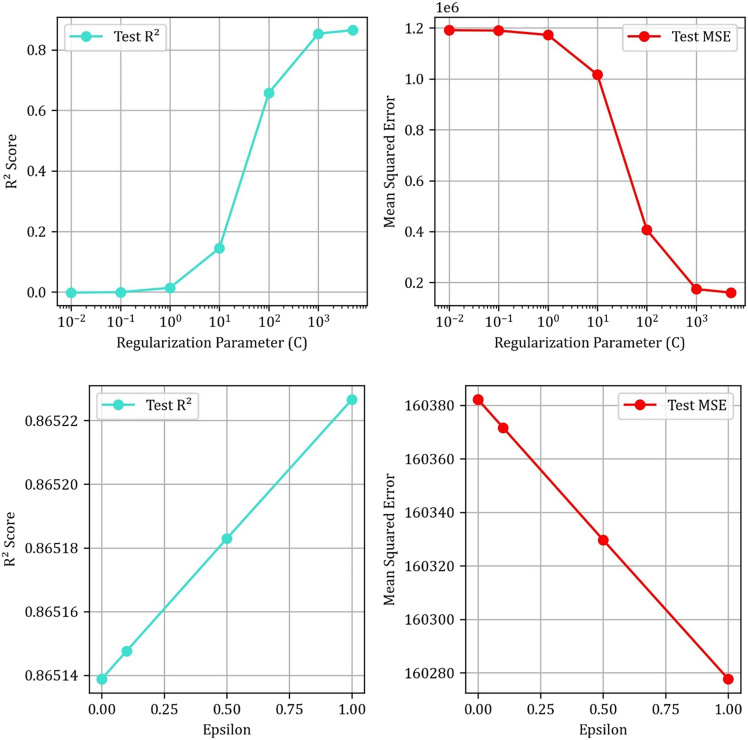
Diagrammatic view of support vector regression (SVR) kernel configuration and parameter selection ranges

SVR results in Figure 5 show the effect of C and ϵ∖epsilonϵ on both R^2^ and MSE. At C = 5000 and ϵ = 5000, the algorithm attained maximum R^2^ ≈ 0.87 and minimized MSE, reflecting the optimal trade-off between margin width and model flexibility. Lower C values widened the margin excessively, causing underfitting, whereas higher C and very small ϵ risked overfitting to noise. The grid search revealed a relatively flat performance plateau near the chosen final parameters, indicating robustness to slight deviations.

**Figure 6:**
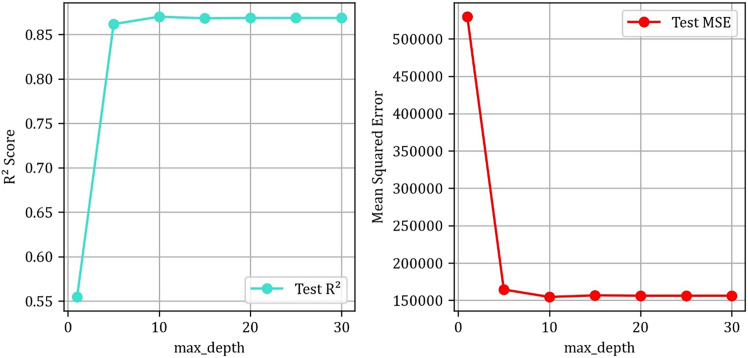
Framework depiction of the random forest regressor with depth and tree count adjustment scheme

Figure 6 demonstrates the effect of maximum depth in random forest models. Depth 10 yielded the best performance (R^2^ ∼0.87), capturing sufficient feature interaction without extending into noise-fitting territory. Shallower depths (≤5) reduced predictive capacity due to insufficient splitting, while depths beyond 15 increased MSE by introducing variance. The final estimators = 100 ensured averaging stability while keeping computational cost moderate.

**Figure 7:**
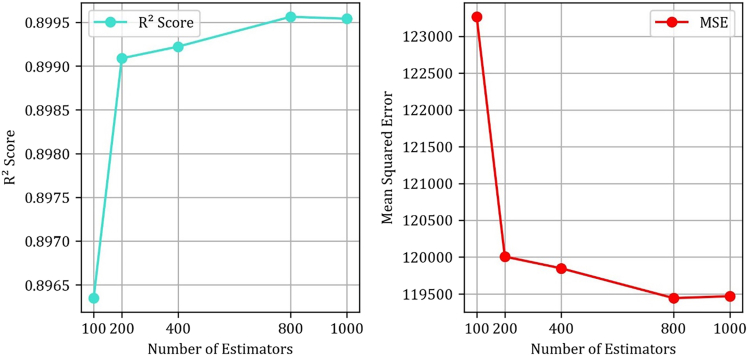
Structural overview of gradient boosting model setup and estimator-learning rate adjustment process

In Figure 7, gradient boosting performance scales with n_estimators. At n_estimators = 800 and learning rate = 0.1, the model reached R^2^ > 0.9 with minimal MSE, balancing depth of boosting against potential overfitting. Smaller ensembles (≤200) produced slower error reduction and reduced fit quality, while excessive ensembles at constant learning rate offered no notable benefit—confirming diminishing returns beyond the optimal.

**Figure 8:**
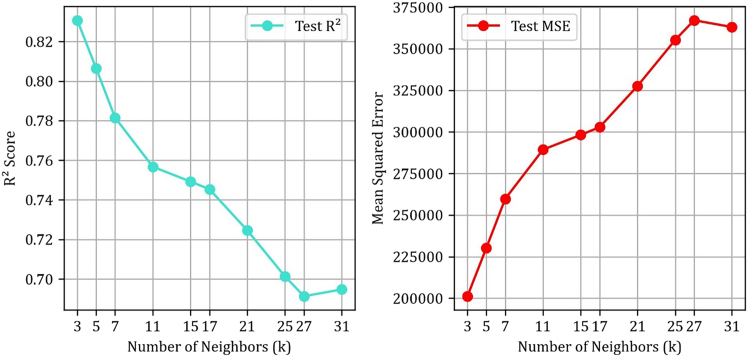
Illustration of k-nearest neighbors (KNN) regressor configuration and neighbor count selection approach

Figure 8 shows the trade-off between number of neighbors and model smoothness. Performance peaked at *n* = 3, yielding R^2^ ≈ 0.83 and low MSE, where the algorithm captured local similarity without being diluted by distant points. Larger neighbor counts (>10) reduced sensitivity to local structure, diminishing predictive precision.

**Figure 9:**
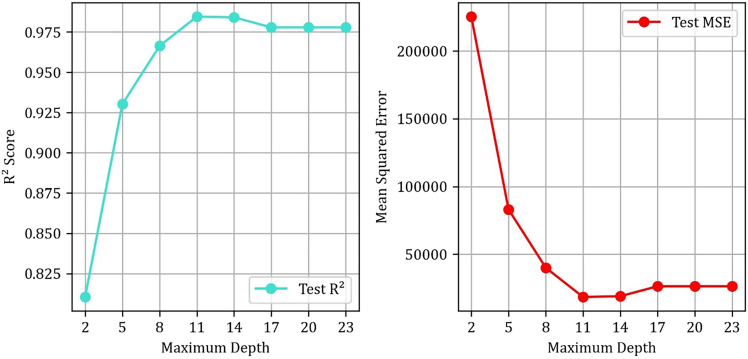
Architectural schematic of decision tree regressor and maximum depth configuration protocol

Decision tree performance in Figure 9 peaks at max_depth = 11 (R^2^ ∼0.98). Shallow trees (<6) underfit, missing complex nonlinear interactions, while deeper trees (>18) increased MSE due to variance inflation. The chosen depth ensures capturing key splits without saturating leaf purity on small subgroups, thus producing a balanced structure.

**Figure 10:**
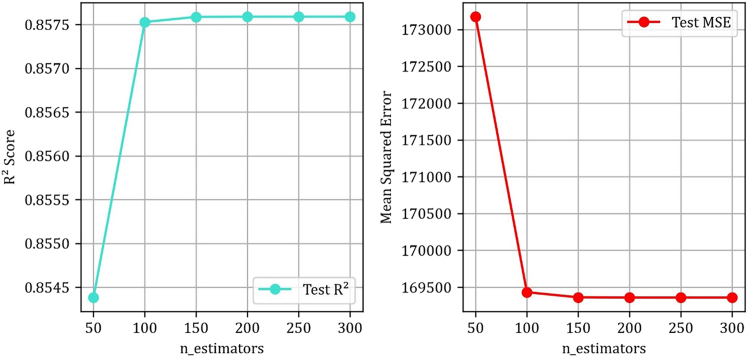
XGBoost regressor design diagram with estimator and learning rate optimization path

Figure 10 reveals that at n_estimators = 250 and learning rate = 0.1, XGBoost achieves stable high performance (R^2^ ≈ 0.86, low MSE). Lower learning rates slowed convergence, while higher rates risked overshooting optimal minima. The optimal ensemble size matched the dataset scale, preventing overfit while maximizing boosting iterations.

**Figure 11:**
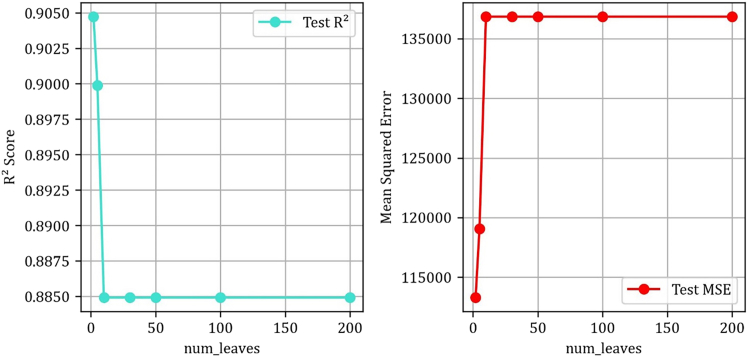
LightGBM regressor structure visualization with leaf number and tree count adjustment plan

LightGBM results (Figure 11) indicate that 2 leaves with n_estimators = 100 and learning rate = 0.1 yield well-generalized fits with R^2^ near 0.9. Increasing leaf count boosted variance and reduced stability, while fewer leaves limited model expressiveness. The histogram-based algorithm benefited from small, well-balanced leaf nodes, improving memory efficiency without sacrificing accuracy.

**Figure 12:**
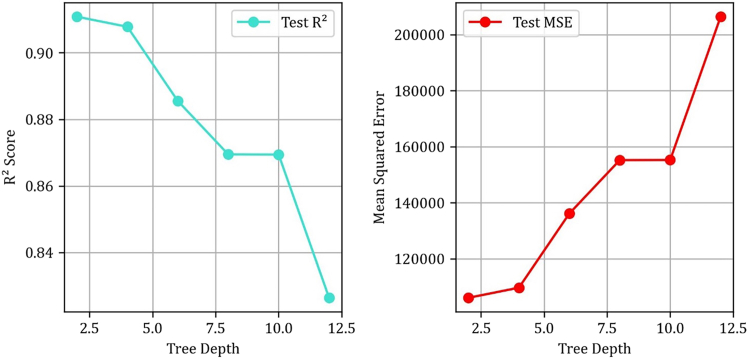
CatBoost regressor configuration overview highlighting tree depth and iteration tuning stages

Figure 12 shows that at tree depth = 2 and learning rate = 0.1, CatBoost obtained R^2^ ≈ 0.89 with low MSE, confirming that shallow oblivious trees can capture categorical feature influences effectively. Depth expansions beyond 6 did not improve accuracy and increased computation time.

Table 1 summarizes the final configurations determined through extensive hyperparameter optimization for all considered algorithms. The ANN and CNN architectures were kept relatively compact to balance generalization and computational efficiency, with carefully selected neuron counts and activation functions to ensure convergence within 2500 iterations. Regularization-based regression models demonstrated sensitivity to α values, converging at moderate penalties to avoid both under- and over-regularization. Ensemble tree-based algorithms such as random forest, gradient boosting, XGBoost, LightGBM, and CatBoost achieved optimal performance with moderate depths and estimator counts, reflecting the dataset’s complexity without excessive variance. Kernel and nearest-neighbor models settled on flexible but not overly permissive parameter values—SVR with large C and ϵ for tolerance, and KNN with only three neighbors to capture local relationships. Collectively, these tuned parameters form a robust baseline, aligning model complexity with dataset scale and ensuring reproducible high-accuracy predictions in subsequent evaluation stages.

**Table 1:** Consolidated summary of final architectures and tuned hyperparameters for all evaluated models

Model	Architecture/Key Parameters	Final Tuned Values
ANN	3 layers (32, 8, 1 neurons), max iterations: 2500, transfer: ReLU (hidden), linear (output)	as stated
CNN	2 Conv layers (8, 16 filters), 2 FC layers (32 neurons, 1 output), max iterations: 2500, transfer: ReLU (conv & FC), linear (output)	as stated
Linear Regression	linear model	no tuning parameters
Ridge Regression	α (regularization strength)	α = 1.6238
Lasso Regression	α (regularization strength)	α = 1.6238
Elastic Net	α (regularization strength), l1_ratio (mixing parameter)	α = 0.01274, l1_ratio = 0.2
SVR	C (regularization), ε (tube width), kernel, γ (kernel coeff.)	C = 5000, ε = 5000
Random Forest	n_estimators, max_depth	n_estimators = 100, max_depth = 10
Gradient Boosting	n_estimators, learning_rate	n_estimators = 800, learning_rate = 0.1
KNN	n_neighbors	n_neighbors = 3
Decision Tree	max_depth	max_depth = 11
XGBoost	n_estimators, learning_rate	n_estimators = 250, learning_rate = 0.1
LightGBM	best leaves No., n_estimators, learning_rate	leaves = 2, n_estimators = 100, learning_rate = 0.1
CatBoost	tree depth, iterations, learning_rate	depth = 2, iterations = 100, learning_rate = 0.1

### Evaluation of the developed data-driven models

From Table 2, models exhibiting the most balanced performance across training, validation, and test sets are those with high R^2^ values and consistently low MSE/MRD% across subsets. The ANN stands out as the overall best performer, achieving R^2^ = 0.99 (train), 0.99 (validation), and 0.99 (test) with low MSE values and minimal MRD% (train: 1.9%, validation: 2.1%, test: 2.0%). This uniformity reflects a well-generalized fit without sacrificing predictive precision. Decision tree also delivers exceptional test accuracy (R^2^ = 0.985) and very low MRD% (<1.5% across all sets), though its unusually high validation score (0.960) compared to other tree ensembles suggests it captured dataset patterns extremely well. LightGBM shows a respectable performance (R^2^ around 0.9) with balanced error metrics, making it a strong, efficient alternative.

**Table 2:** Comparative summary of training, validation, and test evaluation metrics for all implemented predictive models

Model	Train R^2^	Validation R^2^	Test R^2^	Train MSE	Validation MSE	Test MSE	Train MRD%	Validation MRD%	Test MRD%
Linear Regression	0.9155	0.8343	0.9110	86650.4134	167961.5329	105874.8190	3.76	5.07	5.01
Ridge Regression	0.9122	0.8234	0.9079	90127.1976	178981.5472	109582.7791	3.83	5.24	5.02
Lasso Regression	0.9145	0.8259	0.9090	87716.3825	176442.7072	108273.4077	3.77	5.14	4.95
Elastic Net	0.9123	0.8236	0.9079	90016.3587	178807.1031	109519.2134	3.83	5.24	5.02
Gradient Boosting	0.9997	0.7755	0.8996	258.6069	227613.5442	119442.9284	0.04	5.82	4.64
Random Forest	0.9822	0.7932	0.8686	18221.6997	209669.8690	156210.9266	1.75	5.55	5.24
XGBoost	0.9997	0.7396	0.8576	258.3516	263941.7812	169358.8750	0.03	6.21	5.75
LightGBM	0.9098	0.7769	0.9047	92532.1755	226193.3152	113302.4917	4.26	5.56	4.48
CatBoost	0.9407	0.8203	0.8937	60845.8322	182141.1821	126377.7683	3.56	4.81	4.62
SVR	0.9903	0.8341	0.8652	9922.1608	168177.4065	160277.6796	0.67	5.09	5.56
KNN	0.8640	0.8021	0.8308	139584.4667	200573.8923	201231.6471	5.06	6.34	6.76
Decision Tree	0.9567	0.9577	0.9845	44374.9604	42900.1136	18470.4518	1.43	1.57	0.95
Gaussian Process	0.9525	0.8197	0.9154	48697.9751	182788.4273	100574.0411	2.94	5.35	4.69
ANN	0.9934	0.9912	0.9949	6805.6748	8911.8350	6121.1885	1.14	1.34	1.09
CNN	0.9803	0.9756	0.9765	20223.6992	24730.0312	27927.7617	1.91	2.11	2.00

In contrast, KNN yields relatively lower accuracy with R^2^ values below 0.83 for validation and test and the highest MRD% in the test phase (6.8%), indicating difficulty in capturing the complexity of the energetic materials dataset with a purely instance-based approach. XGBoost’s validation R^2^ (0.740) and test R^2^ (0.858) are also notably lower than those of other ensemble methods, hinting at tuning or model-data mismatch issues. Models like linear, Ridge, Lasso, and elastic net regressors, while stable and moderately accurate (R^2^ ∼0.91 test), are less competitive compared to advanced boosting or neural approaches, reflecting intrinsic limitations in linear relationships for such highly non-linear material properties.

Clear overfitting is observed in gradient boosting, random forest, and XGBoost, each showing perfect or near-perfect train R^2^ (1.000) but significantly lower validation/test scores, coupled with extremely small train MSE (<260) and large validation/test MSE (>119k), indicating poor generalization. SVR also shows signs of overfitting with train R^2^ at 0.990 but a drop to 0.834 (validation) and 0.865 (test). In contrast, ANN maintains high train and validation parity, minimizing this effect. Decision tree’s extremely high scores across all sets, while impressive, require caution since a pure tree can easily memorize training data; however, the sustained test accuracy suggests that in this dataset’s scope, it generalized unusually well. Overall, ANN emerges as the most robust top performer, decision tree as a high-accuracy outlier, gradient boosting/random forest/XGBoost as overfitted, and KNN as the weakest approach.

**Figure 13:**
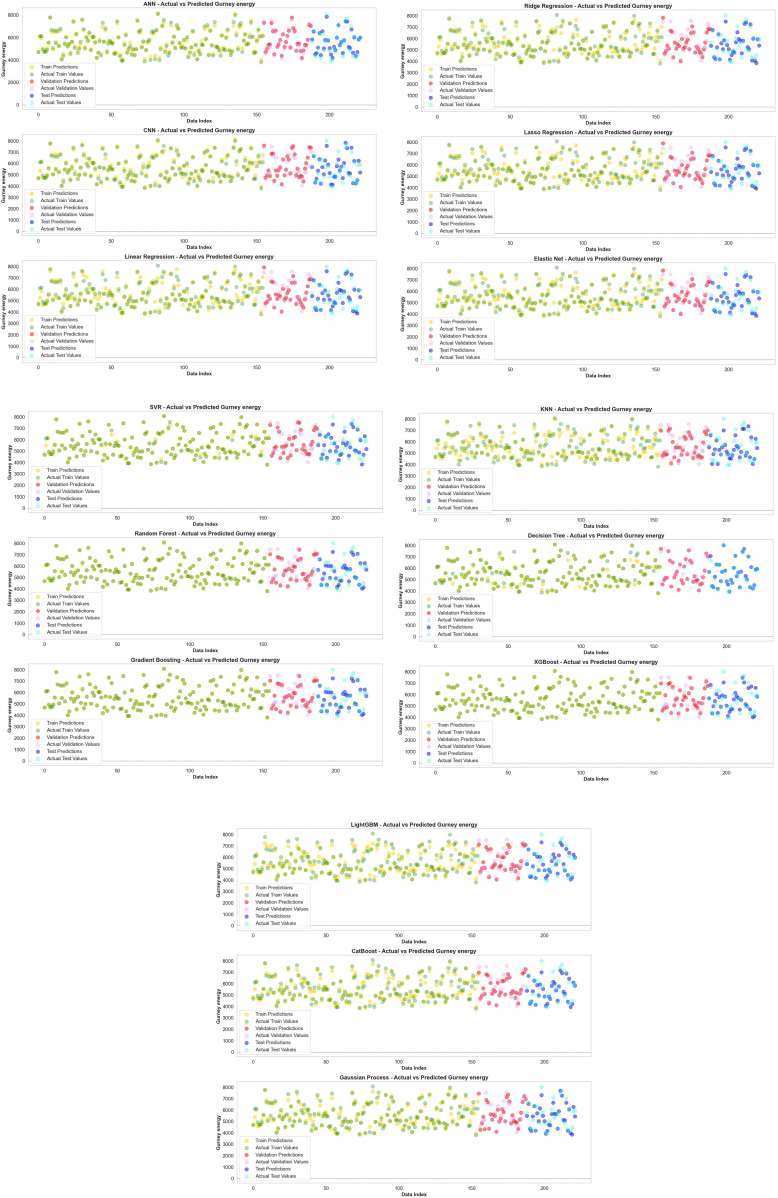
Comparison of actual experimental values and model-predicted outputs across training, validation, and test phases for all implemented algorithms

The comparative plot in Figure 13 illustrates the alignment between actual experimental values and predictions generated by the different ML algorithms across training, validation, and test phases. Models that maintain tightly clustered points along the ideal diagonal line in all phases demonstrate strong generalization, with minimal deviation from true observations. Neural approaches, such as ANN, display consistently high predictive agreement, showing only slight dispersion in test cases, indicating effective learning of complex non-linear relationships within the energetic materials dataset. Decision-tree-based methods also reveal close tracking of actual values, particularly in the test phase, suggesting that judicious depth and parameter selection enabled them to capture key structural patterns without excessive loss in generalization.

Conversely, certain ensemble learners and non-parametric methods exhibit broader scatter, particularly in validation and test plots, reflecting sensitivity to unseen data. Instance-based models such as KNN show noticeable deviations from the diagonal, signifying reduced adaptability when encountering inputs outside the immediate neighborhood of training examples. Boosting algorithms, while often fitting training data exceptionally well, display more spread in validation and test predictions, a clear visual indication of overfitting tendencies. The overall distribution patterns in Figure 13 reinforce that models with balanced complexity and regularization achieve the most reliable phase-to-phase predictive consistency, while overly rigid or highly adaptive learners risk performance drops when moving beyond their training domain.

**Figure 14:**
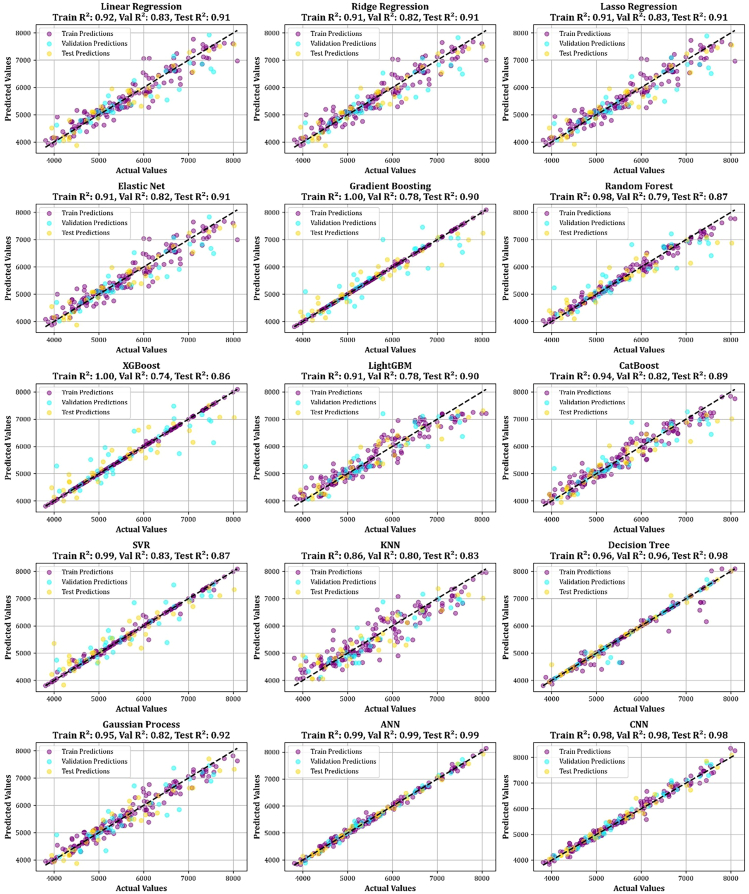
Crossplots of actual versus predicted output values for all implemented machine learning models

The crossplots in Figure 14 provide a visual assessment of how closely each model’s predictions align with the actual output values, effectively serving as a diagnostic of predictive accuracy and generalization. Models producing scatter points clustered tightly along the 1:1 reference line indicate minimal systematic error and strong agreement between predicted and measured values. Neural networks, particularly those with optimized architectures, show dense diagonal alignment across all phases, reflecting their ability to capture complex non-linear dependencies in the energetic materials dataset. Similarly, well-tuned decision-tree-based models exhibit high fidelity in test predictions, with only minor deviations, underscoring their effectiveness in exploiting hierarchical feature interactions.

In contrast, certain algorithms present wider dispersion from the ideal line, especially in the validation and test sets, signaling reduced generalization or sensitivity to data variance. Instance-based approaches demonstrate this clearly, where prediction quality depends heavily on the presence of close training examples, leading to noticeable scatter for less-represented regions of the parameter space. Boosting methods, while often exhibiting near-perfect training fit, show increased deviations in unseen data domains, hinting at overfitting despite their ensemble strength. The overall visual trend from Figure 14 emphasizes that models achieving a balance between complexity and regularization deliver the most stable, phase-to-phase predictive alignment, whereas overly adaptive or rigid models risk performance degradation in out-of-sample predictions.

**Figure 15:**
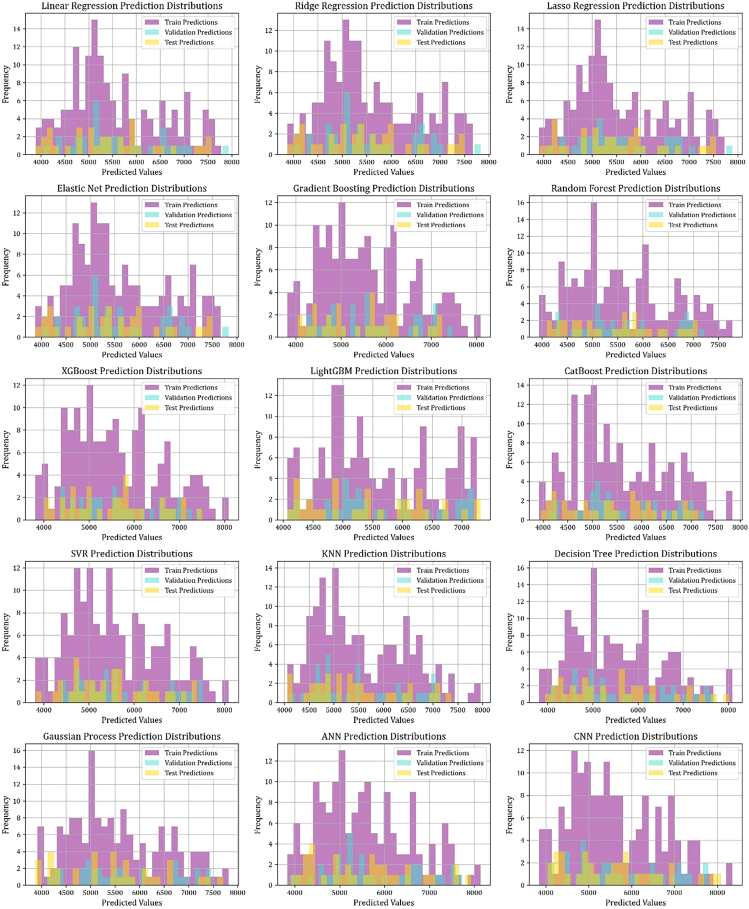
Bar chart distributions of predicted output values across all implemented machine learning models

Figure 15 presents bar chart distributions of predicted values across the implemented ML models, offering insight into how each algorithm maps input features to output magnitudes. The frequency-prediction profiles reveal the density of outputs within specific value ranges, enabling evaluation of model bias toward certain prediction intervals and potential under- or over-representation of extreme outputs. Well-balanced models show relatively uniform coverage across the output spectrum, which indicates their ability to capture both central tendencies and tails of the data distribution. In contrast, models exhibiting sharp frequency peaks or truncated coverage suggest stronger concentration around mean values and possible difficulty extrapolating to higher or lower energetic outputs. This visualization complements phase-wise accuracy assessments by highlighting the statistical spread and distributional behavior of predictions, thereby aiding in diagnosing overfitting, underfitting, or range-limitation tendencies inherent in different learning frameworks.

**Figure 16:**
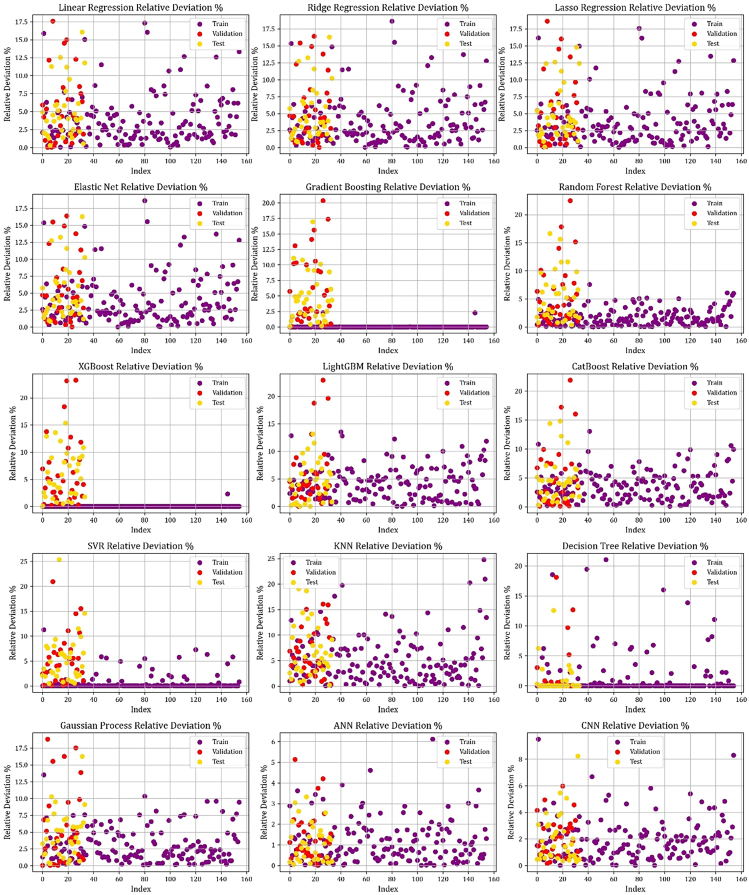
Relative error distribution for each implemented machine learning algorithm across all dataset samples

Figure 16 visualizes the relative error distribution for each data point across different ML algorithms, enabling a detailed assessment of prediction precision at the individual sample level. Models with consistently low relative error bars across the dataset indicate stable predictive capacity and accurate mapping between feature space and output values. Neural approaches, particularly those with optimized architectures and regularization schemes, exhibit narrow error ranges with minimal occurrence of extreme deviations, implying effective learning of both central and peripheral trends in the energetic material properties. Similarly, certain well-tuned tree-based frameworks show compact error profiles, reflecting their ability to partition the input space efficiently while maintaining robust generalization.

Conversely, broader error fluctuations in some methods point to sensitivity toward specific sample compositions or insufficient capture of complex feature interactions. Instance-based algorithms display distinct peaks in error magnitude, often corresponding to data points that are distant from closest training neighbors, highlighting their dependence on localized similarity rather than global trends. Ensemble boosting models, while achieving exceptional fits in training phases, reveal greater spread in relative errors for unseen data, an indication of overfitting, where model flexibility leads to exaggerated responses outside the training domain. The patterns in Figure 16 underscore that algorithms maintaining balanced complexity and effective error regularization deliver the most uniform and reliable point-wise performance across diverse energetic material configurations.

**Figure 17:**
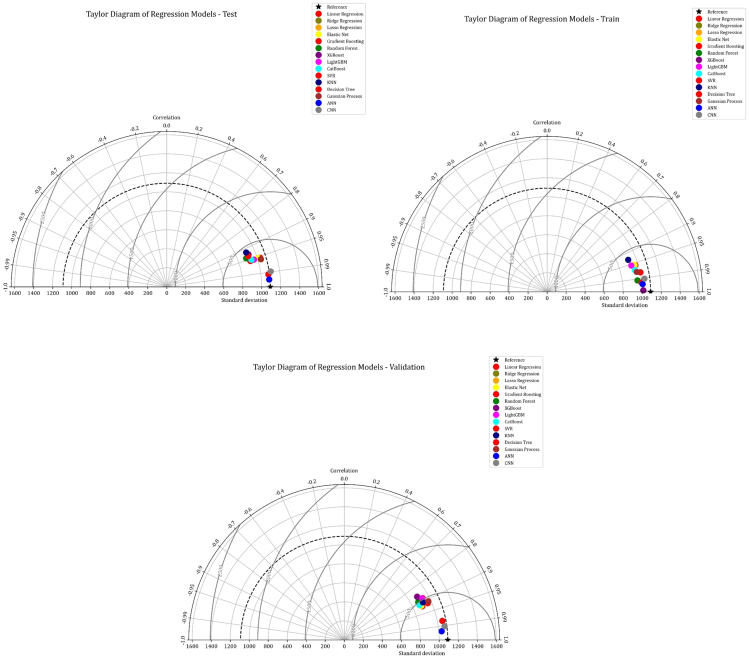
Taylor diagram comparing correlation, standard deviation, and RMSD metrics for all algorithms in training, validation, and test phases

Figure 17 presents a Taylor diagram summarizing the comparative performance of all ML algorithms across training, validation, and test phases by simultaneously depicting correlation coefficients, root-mean-square deviations (RMSD), and standard deviations. Points situated closer to the reference marker with high correlation and low RMSD represent models that closely replicate the observed data patterns while maintaining minimal error. The spatial separation between phases for each algorithm reveals their generalization capacity, with smaller radial shifts indicating consistent predictive behavior across unseen datasets. This multi-metric visualization effectively condenses predictive accuracy, variability reproduction, and overall skill into a single framework, enabling a clear scientific comparison of model robustness and stability.

**Figure 18:**
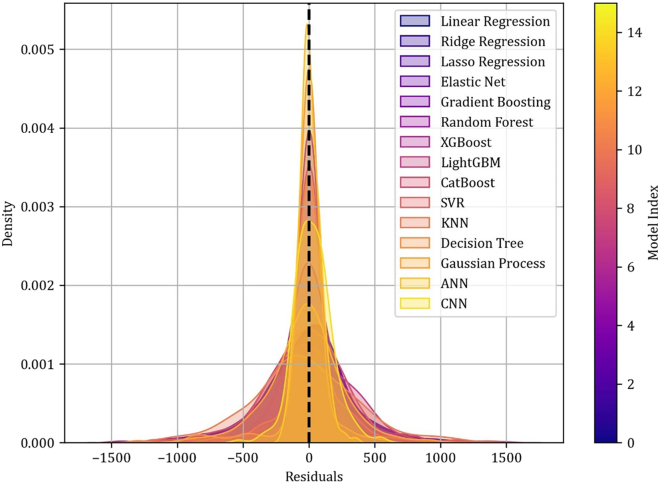
Residual density plots for all implemented machine learning algorithms

Figure 18 displays residual density plots for the implemented ML algorithms, offering a statistical view of the distribution of prediction errors relative to the true values. In these plots, a sharp, symmetric peak centered around zero indicates that most residuals are small and evenly distributed, reflecting balanced model performance without systematic bias. Broader, flatter curves suggest greater variability in residuals, potentially due to underfitting or high variance, while skewed distributions can reveal directional bias in predictions. By examining the spread and symmetry of each curve, this visualization enables a direct comparison of how effectively different models capture the underlying trends of the dataset and how tightly their predictions cluster around the actual values.

**Figure 19:**
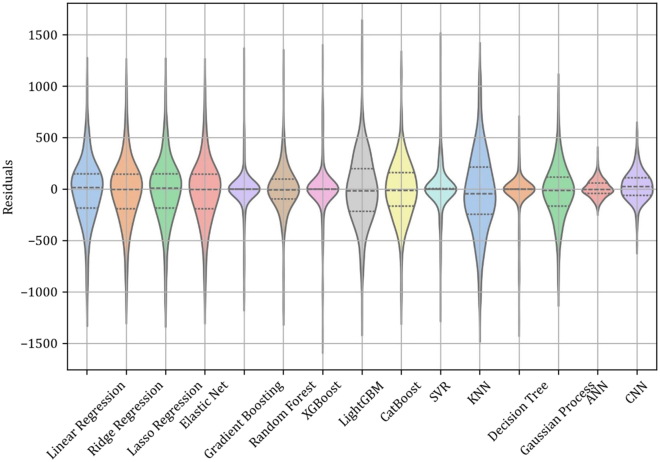
Violin plots illustrating residual distributions for all implemented machine learning algorithms

Figure 19 presents violin plots of residuals for the implemented ML algorithms, combining boxplot elements with kernel density estimation to provide both statistical summaries and the full distribution shape of prediction errors. The width of each violin reflects the relative frequency of residual values at different magnitudes, while the embedded boxplot conveys median, interquartile range, and outlier presence. This visualization enables simultaneous inspection of central tendency, variability, and distribution symmetry, making it straightforward to compare how different algorithms handle error dispersion and whether their residual patterns are concentrated, skewed, or widely spread within the dataset.

**Figure 20:**
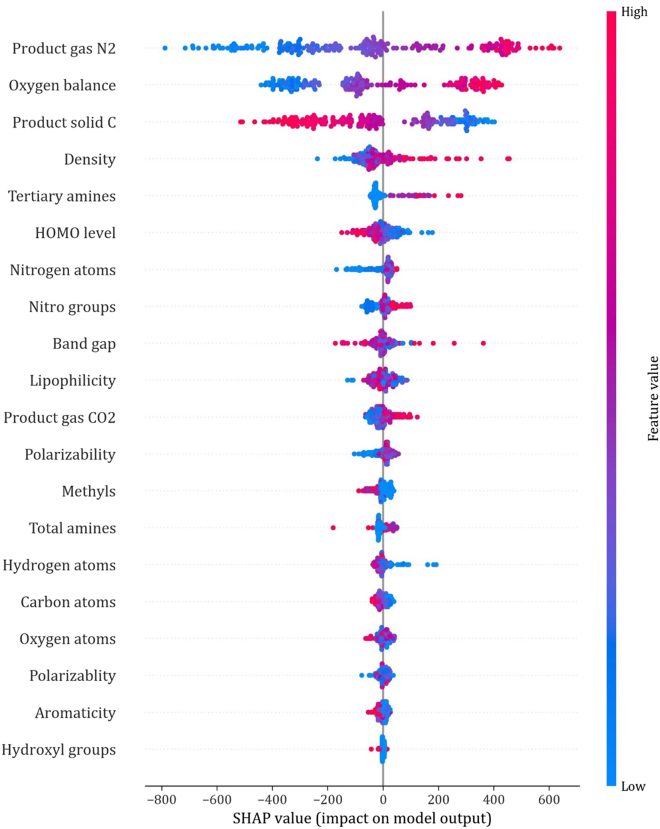
Ranked feature importance with color-coded value indicators for all predictive model inputs

Figure 20 provides a ranked visualization of feature importance for predicting the Gurney energy of energetic materials, arranged from the most to the least impactful input variables. The ranking is derived from model-based feature attribution methods, such as permutation importance or SHAP (shapley additive explanations), which quantify how much variance in the target variable can be explained by each feature. In this representation, the horizontal positioning reflects the magnitude and sign of the feature’s contribution, while the color coding links these contributions to the original feature values—red for high values and blue for low values. This integrated layout allows simultaneous interpretation of both the relative importance and the directional influence of each predictor.

From a scientific standpoint, the top-ranked feature, produced gas N_2_, shows the most significant direct effect on Gurney energy predictions. Nitrogen gas production is critical in detonation physics because gaseous products contribute directly to expansion work, which influences acceleration imparted to the detonation products and hence the calculated Gurney energy. In the diagram, red points (high N_2_ production) being located on the positive side indicate that greater nitrogen output correlates with higher predicted Gurney energy. This aligns with thermochemical principles, where molar gas production, especially non-condensable species, increases detonation pressure and velocity, thereby enhancing the mechanical energy available.

OB emerges as the second most influential factor, with its red points also on the positive side, signifying a direct contribution. OB represents the stoichiometric relationship between the oxygen content in the molecule and the amount required for complete oxidation of carbon and hydrogen. A near-zero or slightly positive OB often promotes optimal conversion of energetic material into gaseous products like CO_2_ and H_2_O while minimizing solid carbon formation. Thus, higher OB values typically translate into improved energy release, driven by increased gaseous output, which the model captures as a positive correlation with Gurney energy.

In contrast, produced solid carbon © ranks third but exhibits an inverse relationship—red points are positioned on the negative side of the diagram. Solid carbon is a condensable detonation product that contributes minimally to expansion work compared to gases and can even impair performance by lowering the effective molar gas fraction. Thermodynamically, increased solid carbon yield implies incomplete oxidation or fuel-rich compositions, where potential chemical energy is locked in condensed phases rather than available for gas expansion. The model’s interpretation reflects this physics: higher solid carbon output reduces the predicted Gurney energy, consistent with the negative contribution visualized in the feature importance ranking. This inverse trend highlights how balanced composition—favoring gaseous over solid products—remains a dominant determinant of kinetic energy transfer in detonations.

This study successfully established a robust ML framework to predict the Gurney energy (EG) of energetic materials utilizing a comprehensive dataset of 222 compounds characterized by intrinsic physicochemical descriptors. By demonstrating that the artificial neural network (ANN) serves as the optimal predictive model, achieving exceptional accuracy with an R2 of 0.99 and MRD <1.5%, this work provides profound practical implications for the energetic materials sector. This high-fidelity, data-driven approach enables rapid in silico screening and iterative formulation design, significantly reducing the massive experimental effort, safety risks, and resource expenditures traditionally required to evaluate explosives. Furthermore, the identification of produced N2 gas, OB, and solid Cyield as the most critical predictive features successfully grounds the computational model in fundamental detonation thermodynamics. However, model generalization remains inherently constrained by the historical chemical space of the training data and the approximation errors propagated from the empirical Kamlet-Jacobs thermochemical inputs.

### Limitations of the study

Despite their robust predictive performance, these ML models possess inherent limitations. First, while the dataset encompasses 222 well-characterized compounds, its chemical space is bounded by historically synthesized materials; extrapolating predictions to fundamentally unprecedented energetic backbones should be approached with caution. Second, the reliance on derived thermochemical parameters propagates any approximation errors and systematic biases inherent to the empirical Kamlet-Jacobs H_2_OCO_2_ equilibrium framework. Furthermore, because the models are designed to evaluate pure molecules based on intrinsic physicochemical properties, they explicitly exclude process-dependent variables such as microstructural morphology, kinetic reaction zone thickness, and confinement conditions. Consequently, the predictions represent idealized theoretical maximum outputs that require eventual formulation-specific experimental validation.

To address these constraints, future research should integrate higher-fidelity quantum mechanical and molecular dynamics simulations to enrich the descriptor sets and strengthen their physical grounding. Expanding training datasets to encompass energetic classes, such as green propellants and nano-engineered composites, will further broaden model applicability. Ultimately, developing hybrid frameworks that combine mechanistic detonation models with ML algorithms will enhance interpretability while preserving predictive power, paving the way toward universally robust energetic estimation tools.

## Resource availability

### Lead contact

Requests for further information and resources should be directed to and will be fulfilled by the lead contact, Alireza Baghban (alireza_baghban@alumni.ut.ac.ir).

### Materials availability

This study did not generate new biological materials or unique reagents.

### Data and code availability

• Data: The curated and standardized dataset of energetic materials used for Gurney energy prediction, including molecular descriptors, thermodynamic properties, and experimental Gurney energy values, is accessible from the lead contact (Alireza Baghban) upon a valid academic inquiry.
• Code: All original code for data preprocessing, feature engineering, ML model development, hyperparameter optimization, and quantitative analysis is accessible from the lead contact (Alireza Baghban) upon a valid academic inquiry.
• Other items: Any additional information, protocols, or analytical methods required to replicate the findings reported in this paper, including feature importance analysis workflows and model evaluation procedures, are accessible from the lead contact (Alireza Baghban) upon a valid academic inquiry.

## Acknowledgments

This work was supported and funded by the Deanship of Scientific Research at Imam Mohammad Ibn Saud Islamic University (IMSIU) under grant no. IMSIU-DDRSP2602.

## Author contributions

Writing – review and editing, validation, supervision, resources, project administration, investigation, formal analysis, conceptualization, M.D.; writing – review and editing, validation, supervision, resources, project administration, investigation, formal analysis, conceptualization, R.H.; writing – review and editing, writing – original draft, visualization, validation, supervision, software, resources, project administration, methodology, investigation, formal analysis, data curation, conceptualization, A.B.

## Declaration of interests

The authors declare no competing interests.

## Declaration of generative AI and AI-assisted technologies in the writing process

During the preparation of this article, the authors used AI assisted tools including ChatGPT, Gemeni, and Grammarly to assist in improving the writing and grammars. After applying aforementioned tools, the authors reviewed and edited the content and take full responsibility for the content of the published article.

## STAR★Methods

### Key resources table

**Table:** 

REAGENT or RESOURCE	SOURCE	IDENTIFIER
**Deposited data**		

Utilized data of Energetic Materials	Liu et al.^19^	

**Software and algorithms**		

Python (v3.8 or higher)	Python Software Foundation	RRID: SCR_008394
Scikit-learn	Pedregosa et al.^24^	RRID: SCR_002577
Pandas	The pandas development team	RRID: SCR_018337
Numpy	Harris et al.^25^	RRID: SCR_008633
Scipy	Virtanen et al.^26^	RRID: SCR_008058
SHAP (shapley Additive explanations)	Lundberg et al.^27^	https://github.com/slundberg/shap
Matplotlib	Hunter^28^	RRID: SCR_008624
Seaborn	Waskom et al.^29^	RRID: SCR_018132

### Experimental model and study participant details

Omitted as our study does not involve biological models.

### Method details

#### Machine learning selection and background

In recent years, machine learning (ML) has emerged as a powerful paradigm for predicting the thermochemical and detonation properties of energetic materials, offering a rapid alternative to computationally expensive quantum mechanical simulations and hazardous experimental testing.^30^^,^^31^^,^^32^^,^^33^^,^^34^ Rather than relying on a single modeling approach, this study employs a diverse suite of fifteen distinct algorithms to ensure a robust comparative analysis. The selection of these algorithms was strategically driven by the characteristics of the dataset, the need to capture complex nonlinear relationships, and the varying requirements for model interpretability. Detailed mathematical formulations and theoretical backgrounds for all the employed methods are provided in the Supplementary Information (Section S1).

The first category of selected models includes traditional and regularized linear approaches (Linear Regression, Ridge, Lasso, and Elastic Net), alongside foundational non-parametric methods such as Decision Trees and K-Nearest Neighbors (K-NN). These algorithms were chosen primarily for their high interpretability and efficiency. Regularized linear models are particularly well-suited for datasets where input descriptors may exhibit multicollinearity, allowing for feature selection and the prevention of overfitting. Conversely, to capture the highly complex and nonlinear physicochemical interactions governing the Gurney energy, we selected powerful kernel-based methods—namely Support Vector Regression (SVR) and Gaussian Processes, as well as advanced tree-based ensemble methods. Ensemble algorithms, including Random Forest, Gradient Boosting, XGBoost, LightGBM, and CatBoost, were prioritized due to their proven prior success in modeling energetic materials, their robustness against outliers, and their exceptional performance on structured, tabular datasets without requiring extensive data scaling.

Finally, deep learning architectures, specifically Artificial Neural Networks (ANN) and Convolutional Neural Networks (CNN), were integrated into the comparative framework. While these models often require larger dataset sizes and function as “black boxes” with lower interpretability, they possess an unparalleled capacity for hierarchical feature extraction and modeling highly intricate, high-dimensional data spaces. By evaluating this comprehensive spectrum of algorithms, from highly interpretable linear models to highly complex deep learning networks, this study identifies the optimal balance between predictive accuracy, computational efficiency, and physical interpretability for estimating the Gurney energy of explosives.

#### Dataset introduction

Extensive experimental work has been reported in the literature on the impact sensitivity and detonation performance of energetic materials, establishing standardized datasets for hundreds of compounds.^2^^,^^35^ In the present dataset, which encompasses 222 energetic compounds, the impact sensitivity values (H_50_) originate from the ERL Type 12 method using a 2.5 kg drop-weight.^1^^,^^35^ Theoretical detonation parameters—including velocity (D), pressure (P), and energy (E)—were derived utilizing the empirical Kamlet–Jacobs (KJ) equations,^1^ The KJ model relies on the H_2_O–CO_2_ equilibrium assumption, dictating the hierarchy of reaction products to calculate the fundamental thermochemical parameters: moles of gaseous products per gram (N), mean molecular weight of products (M), heat of detonation (Q), and crystalline density (ρ). In the present study, we used data gathered from the Liu et al.^19^

To construct a robust feature space for the ML models, a multidimensional set of molecular and physicochemical descriptors was generated using cheminformatics analysis and the RDKit toolkit processing SMILES strings. These input features encompass thermodynamic and macroscopic properties (oxygen balance, density), electronic attributes (HOMO level, band gap, polarizability), and elemental composition. Furthermore, structural characteristics, such as aromaticity, lipophilicity, and the exact counts of specific functional groups (nitro, tertiary amines, hydroxyls, and methyls), were extracted. The predicted detonation products (product gas CO_2_, solid C, gas N_2_) were also computed, creating a comprehensive representation of each pure compound.

Prior to modeling, rigorous dataset standardization was performed to improve the numerical stability and convergence of the ML algorithms. The raw descriptor values were transformed onto the interval [-1,1]using the *Z* score standardization method, where each value X_i_was centered by its mean μand scaled by its standard deviation σ. This preprocessing guarantees that all features contribute comparably to the learning process, eliminating bias introduced by disparate measurement scales. Finally, the dataset was partitioned into a training set (80%) and an independent test set (20%) to ensure an unbiased evaluation of predictive performance.

#### Descriptor selection criteria and model scope

The inclusion criteria for the selected descriptors were strictly grounded in fundamental energetic material theory, ensuring physical interpretability rather than relying solely on data-driven metrics. The Gurney energy (E_G_), or Gurney velocity ($\sqrt{2E_{G}}$), dictates the kinetic energy transferred to a confining casing. Consequently, predictors governing detonation thermodynamics are critical; oxygen balance (OB) indicates combustion efficiency, maximizing the exothermic heat (Q), while the predicted volumes of highly stable nitrogen gas (N_2_) and solid carbon (C) respectively represent massive volumetric expansion capabilities and thermodynamic energy losses. Additionally, macroscopic initial density (ρ) drives the Chapman-Jouguet pressure, and electronic descriptors (HOMO, band gap) serve as quantum mechanical proxies for molecular stability and activation energy, explicitly linking molecular structure to macroscopic explosive power.

To maintain the model’s scope as an *a priori* high-throughput screening tool for them, un-synthesized energetic molecules, several variable classes were intentionally excluded. Specifically, formulation-dependent microstructural parameters (particle size, porosity), kinetic variables (reaction zone thickness), and confinement-related engineering factors (casing geometry, initiation pressure) were omitted. Consistent datasets for these parameters across hundreds of diverse compounds are unavailable in the open literature, and they reflect manufacturing variances rather than the fundamental nature of the pure molecule. By isolating intrinsic structure-to-property relationships, the proposed models establish a robust, theoretical baseline for the maximum kinetic energy transfer, serving as a critical first step in the computational design pipeline.

To visualize the variance, data coverage, and underlying relationships within this multidimensional feature space, a pairwise scatterplot matrix was generated (Figure S1). The diagonal axes display the kernel density estimations (KDE) for individual parameters, revealing the frequency distributions and potential clustering of physicochemical traits across the 222 energetic compounds. The off-diagonal scatterplots illustrate the inter-feature correlations, highlighting both distinct functional groupings and instances of multicollinearity (such as the stoichiometric relationships between atomic compositions and product gases) that the subsequent ML algorithms must navigate.

Figure S2A presents the outlier detection process applied to the dataset using the Multi-Class Outlier Detection (MCOD) approach. This method examines the local and global data structure to identify points that deviate significantly from the typical distribution of energetic material parameters. The plot visually maps all samples, enabling the detection of unusual patterns or extreme values that could distort model training or evaluation. The MCOD assessment in Figure S2A shows that the dataset structure is well-balanced, with no pronounced anomalies or irregular clusters, indicating a clean and reliable feature space for subsequent ML modeling.

Figure S2B complements this quality check by displaying boxplots of the output variables, summarizing their statistical dispersion, median, and interquartile range. The boxplot visualization clearly indicates symmetric or near-symmetric distributions without excessive variability or skewness, and the absence of extreme outliers aligns with the findings from Figure S2A. The consistent upper and lower whisker spans demonstrate that all measured energetic material properties fall within expected ranges derived from experimental and computational protocols. Overall, the outcomes from both subfigures reinforce the integrity and suitability of the dataset for robust predictive analysis, confirming that preprocessing and feature compilation have preserved a sound data foundation.

#### Sensitivity analysis

Figure S3 displays the Pearson correlation coefficient heatmap for the entire dataset, covering all parameters in pairwise (2-v-2) comparisons. The coefficients range from −1 to +1, where values near +1 indicate strong positive linear association between the variables and values near −1 indicate strong negative linear association, while values close to zero reflect negligible correlation.^36^^,^^37^^,^^38^ This visualization condenses the complex relationships among structural, compositional, and thermochemical descriptors into a clear color-coded matrix, allowing rapid identification of parameter interdependencies. By mapping every variable against all others, the heatmap serves as both a diagnostic tool for feature selection and a scientific reference for understanding underlying physical trends.

Inspection of the correlations with the output parameters reveals that certain features exert a consistently positive influence. Higher oxygen content in molecular composition shows a positive correlation with energetic outputs, likely due to its role in complete oxidation reactions enhancing detonation energy and velocity. Crystal density also correlates positively, reflecting the physical effect of compact molecular packing on energy propagation. Band gap and polarizability contribute positively, pointing to the interplay between electronic structure and detonation performance. Among reaction product variables, the amounts of N_2_, atomic oxygen (O), and produced CO_2_ are positively associated with model outputs, consistent with the thermochemical pathways in the H_2_O–CO_2_ equilibrium assumption used in detonation parameter calculations.

Conversely, several parameters exhibit negative correlation with the outputs, indicating that as these values increase, the modeled detonation performance or sensitivity tends to decrease. This group includes descriptors linked to excess hydrogen, higher C/H ratios beyond optimal oxidation balance, and molecular features associated with inert or stabilizing functional groups. Negative correlations can signal dissipative or non-energetic structural characteristics, which absorb or redirect energy away from rapid detonation. The contrasting correlation patterns across variables underscore the multifactorial nature of energetic material performance, highlighting the importance of balancing compositional and structural factors when engineering new formulations.

Regarding data integrity and feature engineering, a rigorous evaluation of the dataset confirmed the absence of any missing values; consequently, no data points required omission or imputation. Retaining the complete dataset ensures maximum statistical power and prevents the introduction of sampling bias that frequently accompanies data exclusion. Furthermore, to evaluate the relationships between the input features and the target variable, a Pearson correlation matrix was constructed (Figure S3). The correlation analysis reveals that several descriptors exhibit strong linear relationships with the Gurney energy, most notably oxygen balance (r = 0.85), predicted product gas N_2_ (r = 0.79), and predicted product solid C (r = −0.84). Figure S3 also highlights instances of significant inter-feature correlations (multicollinearity), such as the high correlation between the number of nitro groups and oxygen atoms (r = 0.97), and between oxygen balance and product solid C (r = −0.97). Despite these collinearities, no descriptors were omitted based on arbitrary correlation thresholds. The physical and chemical reasoning behind this decision is that each descriptor provides a unique, albeit related, piece of thermodynamic or structural information vital to defining the explosive’s profile. Instead of manual omission, which could inadvertently discard subtle non-linear interactions—the comprehensive feature space was preserved. To mitigate the potential negative impacts of multicollinearity on model robustness and variance, this study specifically employed regularized linear models (Ridge, Lasso, Elastic Net) and advanced tree-based ensembles (Random Forest, XGBoost). These algorithms inherently perform continuous penalization or implicit feature selection during training, thereby maintaining predictive stability and minimizing bias without requiring the explicit removal of descriptors.

#### Modeling evaluation

Prior to model training, a rigorous data quality assessment was conducted to address any potential missing values or outliers. The evaluation confirmed that the dataset was fully complete, containing no missing values across any of the selected physicochemical descriptors or the target Gurney energy. As a result, neither row deletion nor data imputation methods were necessary, ensuring that the empirical integrity of the dataset was perfectly preserved without introducing the biases often associated with synthetic data generation. Additionally, no data points were excluded on the basis of being statistical outliers. In the domain of energetic materials, extreme values often represent compounds or specific structural classes with unique performance characteristics rather than erroneous measurements. Retaining the complete set of data points is crucial for capturing the entire chemical space and structural diversity of the compounds. This inclusive approach significantly enhances the broad applicability and generalizability of the trained ML models, particularly since the employed tree-based ensemble algorithms (such as Random Forest and XGBoost) are inherently robust to data variance and extreme values.

Evaluation of predictive performance is a central stage in data-driven modeling, providing quantitative measures of accuracy, bias, and reliability for each applied ML algorithm. In this study, several standard statistical metrics were employed to comprehensively assess model outputs against experimental or calculated reference values: the coefficient of determination (R^2^), mean squared error (MSE), and mean relative deviation percentage (MRD %). These metrics jointly capture goodness of fit, magnitude of error, and proportional deviation, ensuring balanced interpretation of results across models exhibiting different levels of variance and bias.

R^2^ quantifies the proportion of variance in the dependent variable that is predictable from the independent variables. It is expressed as^39^^,^^40^:

(Equation 1) $$R^{2}=1-\left(\frac{\sum_{i=1}^{n}{\left(y_{i}-\hat{y_{i}}\right)}^{2}}{\sum_{i=1}^{n}{\left(y_{i}-\bar{y}\right)}^{2}}\right)$$

where y_i_ is the actual value, ỳ_i_ is the predicted value, and Ӯ is the mean of the actual values. A value of R^2^ represents a perfect fit, while values close to zero indicate that the model fails to explain the variability of the data.

The mean squared error (MSE) measures the average of the squared differences between predicted and actual values, capturing absolute error magnitude in squared units of the output^41^:

(Equation 2) $$MSE=\left(\frac{1}{n}\right)\sum_{i=1}^{n}{\left(y_{i}-\hat{y_{i}}\right)}^{2}$$

Lower MSE values indicate higher predictive accuracy, though interpretation must consider the scale of target values since MSE is sensitive to large errors.

The mean relative deviation percentage (MRD %) evaluates the average proportional deviation of predictions from actual values, expressed as a percentage for scale-independent interpretation:

(Equation 3) $$MRD\%=\left(\frac{100}{n}\right)\sum_{i=1}^{n}\left|\frac{\left(y_{i}-\hat{y_{i}}\right)}{y_{i}}\right|$$

This metric facilitates direct comparison between models across datasets of varying magnitude and units, reflecting how well predictions align with experimental results relative to the actual measured values. Low MRD % implies close alignment between predicted and actual data and high model reliability.

### Quantification and statistical analysis

All model training, validation, testing, and statistical analyses were conducted utilizing Python and its associated scientific libraries (Scikit-learn, Pandas, NumPy, SciPy). The exact sample size utilized for all statistical evaluations is *n* = 222, which represents the total number of unique energetic compounds. To measure and evaluate model performance, center, dispersion, and precision, we calculated the Coefficient of Determination (*R*^2^), Mean Squared Error (MSE), and Mean Relative Deviation (MRD%). Further statistical dispersion, variance, and correlations were quantified and visualized using relative error distributions, residual density analyses, and Taylor diagrams. Complete statistical details, including exact data partition ratios, specific hyperparameter values, and individual metric scores across all subsets, can be found in the Results section, Tables 1 and 2, and the corresponding figure legends.

## Supplemental information

Document S1. Figures S1–S3, Methods S1 and references
