# Supplementary material for: Machine learning innovations for reliable gurney energy estimation in energetic materials
Source: iScience. 2026 Jun 25;29(7):116536. doi: 10.1016/j.isci.2026.116536 (PMC13378357; doi:10.1016/j.isci.2026.116536)
Supplement: Document S1. Figures S1–S3, Methods S1 and references [file mmc1.pdf]

iScience, Volume *29*

Supplemental information

Machine learning innovations for reliable gurney energy estimation in energetic materials

Mingyue Deng, Raouf Hassan, and Alireza Baghban

Methods S1. Machine learning background

In this part, we shall investigate the introduction of tree-based machine learning algorithms utilized in our study.

Artificial Neural Network

Artificial Neural Networks are computational models inspired by biological neural systems, consisting of interconnected nodes (neurons) organized in layers. Each neuron computes a weighted sum of its inputs and applies a nonlinear activation function, allowing representation of complex nonlinear mappings. The theory of ANNs is grounded in universal function approximation, the capability to approximate any continuous function with sufficient neurons and layers. Training involves weight optimization via algorithms like stochastic gradient descent with backpropagation. Philosophically, ANNs embody adaptive learning from data, automatically discovering hierarchical feature representations.

Advantages include strong performance in modeling nonlinear and high-dimensional relationships, adaptability to a wide range of problem domains (regression, classification, sequence modeling), and generalization capabilities when properly regularized. Disadvantages include dependence on large datasets for effective learning, sensitivity to hyperparameter settings (learning rate, architecture depth), and comparatively opaque decision processes (black box issue). ANNs can also be computationally expensive, requiring specialized hardware for deep architectures. [1-3]

Convolutional Neural Network

Convolutional Neural Networks are a specialized type of ANN designed for structured grid data such as images. CNNs use convolutional layers to apply learned filters (kernels) across local regions of the input, capturing spatial features hierarchically. Theoretical foundations lie in signal processing and shared-weights architecture, which reduces parameters and exploits spatial stationarity. Pooling layers further condense spatial information, while deeper layers capture increasingly abstract representations. Philosophically, CNNs reflect the principle of local feature composition, building complex patterns from simpler local patterns.

Advantages include high accuracy in tasks involving spatial or temporal locality, ability to automatically learn features without manual engineering, and parameter efficiency due to weight sharing. CNNs dominate fields like computer vision, document analysis, and scenario recognition. Disadvantages include requirement for large labeled datasets, high computational demands (especially for deep CNNs), and reduced performance when spatial structure is weak or irrelevant. CNNs also share the "black box" challenge of interpretability common to deep learning models. [4-7]

Linear Regression

Linear Regression is one of the most fundamental statistical learning methods, based on the assumption of a linear relationship between explanatory variables and the dependent variable. Mathematically, it models the target y as

$$y = \beta_0 + \sum_{i=1}^{p} \beta_i x_i + \varepsilon \tag{1}$$

where βi represents the regression coefficients and ε the error term. Theory-wise, LR applies the method of least squares to minimize the sum of squared residuals, yielding coefficient estimates with well-defined statistical properties under Gauss–Markov assumptions. The philosophy of LR emphasizes simplicity, interpretability, and the notion that many real systems can be approximated linearly over limited domains. It remains widely used in statistics, econometrics, and physical sciences due to its analytical transparency.

LR's main advantage lies in its interpretability, computational efficiency, and well-established inferential framework — allowing clear insight into feature effects and statistical significance. Applications span predictive modeling, trend analysis, and hypothesis testing in fields from engineering to social sciences. However, LR assumes linearity, independence, homoscedasticity, and absence of multicollinearity, making it prone to poor performance under strong nonlinearities or in the presence of correlated features. Sensitivity to outliers and potential overfitting in high-dimensional settings without regularization are further limitations. [8-11]

Ridge Regression

Ridge Regression extends the ordinary least squares framework by adding an L2-norm penalty to regression coefficients, formally minimizing:

$$Loss = \sum_{i=1}^{n} (y_i - y_i)^2 + \lambda \sum_{j=1}^{n} \beta_j^2 \qquad (2)$$

where λ is the regularization parameter. This shrinkage of coefficients combats multicollinearity and reduces variance by introducing bias in a controlled manner. Philosophically, Ridge Regression reflects the principle of trading small increases in bias for significant variance reduction, improving generalization without drastically altering the structure of the model. It is grounded in Tikhonov regularization from numerical analysis.

Advantages include robustness to correlated predictors, stability in high-dimensional settings, and prevention of coefficient blow-up when predictors are nearly collinear. Ridge regression is especially suitable for problems with many small/medium effects spread across predictors. Disadvantages arise from the fact that coefficients are shrunk but never driven exactly to zero — thus Ridge cannot perform variable selection. Moreover, the optimal λ requires careful tuning, and performance may still degrade if the underlying relationship deviates sharply from linearity. [12, 13]

Lasso Regression

Lasso regression modifies the least squares objective by imposing an L1 norm penalty:

$$Loss = \sum_{i=1}^{n} (y_i - y_i)^2 + \lambda \sum_{j=1}^{n} \left| \beta_j \right| \qquad (3)$$

Unlike Ridge, the L^1 penalty can drive some coefficients exactly to zero, performing feature selection alongside regularization. Theoretically, Lasso is related to convex optimization and the geometry of the L^1-ball, where the sharp corners encourage sparsity. Philosophically, Lasso embodies parsimony — the idea that simpler models with fewer predictors often generalize better and are easier to interpret, especially for large feature spaces.

Advantages include automatic variable selection, interpretability through sparsely, and effective handling of high-dimensional datasets. It is widely applied in genomics, image compression, and sparse signal recovery. Disadvantages include instability when predictors are strongly correlated — Lasso may select one arbitrarily and discard others — and bias introduced by excessive shrinkage. Furthermore, like Ridge, its performance depends on the proper choice of λ\lambdaλ, and in cases where all variables carry small but meaningful contributions, Lasso may be too aggressive in exclusion. [14, 15]

Elastic Net

Elastic Net combines L_1 and L_2 penalties in the objective function:

$$Loss = \sum_{i=1}^{n} (y_i - \bar{y}) + \lambda_t \sum_{j=1}^{n} \left|\beta_j\right| + \lambda_2 \sum_{j=1}^{n} \beta_j^2 \qquad (4)$$

Here, α balances the Lasso-style sparsity and Ridge-style shrinkage. This hybrid approach addresses limitations of both methods, especially in cases of highly correlated predictors, where Lasso alone may struggle. The philosophy behind EN is integrative — uniting the bias–variance trade-off management of Ridge with the variable selection capability of Lasso to achieve both stability and sparsity.

Advantages include strong performance in “grouped variable” scenarios, better feature selection stability under multicollinearity, and applicability to high-dimensional problems. EN often outperforms pure Lasso or Ridge when the data structure supports mixed penalties. Disadvantages are the need to tune two hyperparameters (λ and α), added computational cost compared to simple methods, and residual bias from double shrinkage. Moreover, like its parent methods, EN remains limited in representing complex nonlinear relationships without transformation or kernel extension. [16-18].

Random forest

Random Forest is an ensemble learning method that constructs a multitude of decision trees and aggregates their predictions via majority voting (for classification) or averaging (for regression). It uses the principle of bootstrap

aggregating (bagging) and introduces additional randomness by selecting a random subset of features for each split, reducing correlation between trees. The underlying theory is rooted in statistical resampling and variance-reduction concepts: by averaging diverse models, Random Forests achieve stable and generalizable predictions. Philosophically, RF exemplifies the idea that combining many weak or moderately strong learners can outperform a single high-variance model.

Advantages include high predictive accuracy, robustness to overfitting compared to individual decision trees, and the ability to handle large datasets with high dimensionality and mixed data types. RF can naturally estimate feature importance, aiding interpretability in complex datasets. Disadvantages are increased computational cost for very large forests, reduced transparency compared to simple models, and possible underperformance when relationships are heavily linear and easily modeled by simpler algorithms. Additionally, RF can be biased toward features with many possible split points if not corrected. [19, 20]

Gradient Boosting

Gradient Boosting is an ensemble learning technique that builds predictive models sequentially, where each new model attempts to correct the errors of the combined existing models. It typically involves decision trees as base learners, optimizing a differentiable loss function via gradient descent in function space. Formally, it constructs an additive model:

$$F_m(x) = F_{m-1}(x) + \lambda_m h_m(x) \qquad (5)$$

where h_m is the weak learner fitted to the current residuals, and γ_m is the step size determined to minimize the loss. The theory draws from numerical optimization and statistical boosting principles, focusing on stage-wise minimization of prediction error.

Advantages include high predictive accuracy, flexibility to model complex nonlinear relationships, and applicability to diverse loss functions (regression, classification, ranking). GB can handle heterogeneous feature types and interactions effectively without explicit feature engineering. Disadvantages include higher computational cost, sensitivity to hyperparameter tuning, and risk of overfitting if the boosting process is not

properly regularized. Additionally, interpretability may be reduced compared to simpler regression models, especially with a high number of boosting rounds.

K-Nearest Neighbor

K-Nearest Neighbors is a non-parametric, instance-based learning algorithm that predicts outputs based on the average (regression) or majority class (classification) of the k closest training samples according to a distance metric (e.g., Euclidean, Manhattan). It assumes local similarity — that instances close in feature space have similar target values. The theory builds upon metric spaces and neighborhood approximations, with model complexity existing entirely in the training data rather than learned parameters. Philosophically, KNN represents simplicity in machine learning: no explicit training phase, relying on direct pattern matching in the dataset.

Advantages include conceptual simplicity, flexibility in adapting to different distance measures, and potential to capture complex decision boundaries with sufficient data density. It can work well in cases where local relationships dominate. Disadvantages are sensitivity to the choice of k and distance metric, poor scalability to large datasets due to high query-time cost, and performance degradation in high-dimensional spaces (curse of dimensionality). Additionally, KNN's predictions can be heavily influenced by irrelevant or noisy features without feature scaling or selection. [20-22]

eXtreme Gradient Boosting

XGBoost is a highly optimized implementation of gradient boosting that incorporates advanced regularization, parallelization, and efficient handling of sparse data. The core algorithm sequentially builds decision trees, where each tree corrects the residual errors of the ensemble so far, minimizing a chosen loss function via gradient descent in function space. It uses both L1 and L2 regularization on leaf weights, enhancing generalization. The philosophy behind XGBoost centers on computational efficiency and scalability — aiming to bring boosting methods to massive, real-world datasets with minimal training time.

Advantages include exceptional performance in terms of speed and accuracy, built-in management of missing values, and suitability for high-dimensional structured data. XGBoost's regularization tends to reduce overfitting

compared to plain gradient boosting. Disadvantages involve the need for careful hyperparameter tuning, sensitivity to noisy data, and reduced interpretability due to the dense sequence of trees. Model complexity can lead to long inference times in deployment if ensembles are very large. [20, 23]

LightGBM

LightGBM is an optimized gradient boosting framework using tree-based learning, designed for efficiency and scalability on large datasets. Its theoretical foundation is similar to gradient boosting, but it employs a histogram-based algorithm for splitting and a leaf-wise growth strategy with depth constraints, which reduces memory usage and speeds up training significantly. Philosophically, LightGBM focuses on resource-friendly computation that still leverages the strong predictive power of boosting — enabling use in environments with strict memory and time constraints.

Advantages include extremely fast training, low memory consumption, and strong performance in both classification and regression tasks with large datasets. The leaf-wise growth often yields lower loss than level-wise methods given proper parameter control. Disadvantages include potential overfitting in small datasets due to aggressive leaf growth, reduced transparency from the histograms' binning process, and sensitivity to parameter choices for max depth and number of leaves. [24, 25]

Categorial Boosting

CatBoost is a gradient boosting algorithm developed with built-in support for categorical features, using an ordered target statistics encoding that mitigates target leakage. It grows oblivious decision trees (symmetric trees with the same splitting criterion across all leaves at a given depth), enhancing efficiency and reducing overfitting. The theory integrates boosting principles with robust preprocessing for categorical variables, eliminating the need for extensive manual encoding. Philosophically, CatBoost emphasizes automation and bias reduction, making advanced boosting methods more accessible without extensive data cleaning.

Advantages include native handling of categorical variables, fast training with competitive accuracy, and reduced risk of overfitting due to ordered boosting. CatBoost often performs well even with minimal hyperparameter

tuning. Disadvantages involve relatively higher memory requirements for extremely large datasets and potentially slower inference compared to lighter models. Additionally, while it automates encoding, its transformations can be opaque to inexperienced analysts seeking interpretability. [26, 27]

Gaussian Process

Gaussian Process regression is a non-parametric, Bayesian approach to modeling distributions over functions, where any finite set of function values has a joint Gaussian distribution. A GP is fully specified by a mean function and a covariance (kernel) function, the latter encoding assumptions about smoothness, periodicity, or other properties. Predictions are made by conditioning the joint Gaussian distribution on observed data, yielding closed-form posterior means and variances. Philosophically, GP regression emphasizes probabilistic modeling and uncertainty quantification — viewing predictions as distributions rather than single point estimates.

Advantages include flexibility to model complex functions without fixed parameterization, inherent uncertainty estimates for predictions, and easy incorporation of prior knowledge via kernels. GPs are widely used in spatial statistics, surrogate modeling, and Bayesian optimization. Disadvantages include high computational cost, scaling as $O(n^3)$ with dataset size due to matrix inversion, limiting practicality to small or medium datasets. Performance is highly dependent on kernel choice, and tuning multiple hyperparameters can be non-trivial. [28-30]

Decision Tree

A Decision Tree is a tree-structured predictive model where each internal node represents a test on a feature, each branch corresponds to an outcome of the test, and each leaf node assigns a prediction. The theoretical foundation comes from recursive partitioning of the input space, typically selecting splits that maximize information gain (classification) or minimize variance (regression). Algorithms such as CART (Classification and Regression Trees) rely on impurity measures like Gini index or entropy for classification and mean squared error for regression. Philosophically, DTs embody the principle of divide-and-conquer: breaking a complex decision space into smaller, simpler subspaces where predictions are easier.

Advantages include interpretability, the ability to handle mixed data types, and insensitivity to feature scaling. Decision Trees can model nonlinear relationships and feature interactions naturally. Disadvantages involve high variance — small changes in data can produce very different trees — and a tendency to overfit unless pruned or regularized. Moreover, decision trees can be biased toward features with more levels and can struggle with sparse high-dimensional data without ensemble amplification.

Support Vector Regression

Support Vector Regression extends the Support Vector Machine framework to regression tasks, optimizing a function that predicts within a tolerance ϵ from the true target values, while minimizing model complexity. The theory relies on kernel functions to project data into higher-dimensional spaces where linear regression can be performed on transformed features. SVR seeks a “flat” regression line in feature space that lies within the ϵ-tube, using regularization parameter C to control trade-offs between margin width and tolerance violations. Philosophically, SVR is grounded in structural risk minimization — striving for good generalization by balancing training error and model capacity.

Advantages include strong performance on nonlinear problems via kernels, robustness to outliers due to the ϵ-insensitive loss, and flexibility in handling high-dimensional data. Disadvantages include high computational cost for large datasets, difficulty in tuning parameters (C, ϵ, and kernel settings), and reduced scalability compared to tree-based methods. Moreover, interpretability can be limited, particularly with complex kernel transformations. [31, 32]

References

1. Geeitha, S., et al., *Integrating cat boost algorithm with triangulating feature importance to predict survival outcome in recurrent cervical cancer.* Scientific Reports, 2024. **14**(1): p. 19828.

2. Guo, Z., X. Wang, and L. Ge, *Classification prediction model of indoor PM2. 5 concentration using CatBoost algorithm.* Frontiers in Built Environment, 2023. **9**: p. 1207193.

3. Lalwani, P. and G. Ramasamy. *Hybrid Principal Component Analysis Using Boosting Classification Techniques: Categorical Boosting*. Springer.

4. Ramaneswaran, S., et al., *Hybrid inception v3 XGBoost model for acute lymphoblastic leukemia classification.* Computational and Mathematical Methods in Medicine, 2021. **2021**(1): p. 2577375.

5. Agarwal, R., et al., *Hybrid Deep Learning Algorithm-Based Food Recognition and Calorie Estimation.* Journal of Food Processing and Preservation, 2023. **2023**(1): p. 6612302.

6. Itano, F., M.A.d.A.d. Sousa, and E. Del-Moral-Hernandez. *Extending MLP ANN hyper-parameters Optimization by using Genetic Algorithm*. in *2018 International Joint Conference on Neural Networks (IJCNN)*. 2018.

7. Yang, Y., *A machine-learning prediction method of lithium-ion battery life based on charge process for different applications.* Applied Energy, 2021. **292**: p. 116897.

8. Liu, J., Y. Gao, and F. Hu, *A fast network intrusion detection system using adaptive synthetic oversampling and LightGBM.* Computers & Security, 2021. **106**: p. 102289.

9. Kavitha, S., S. Varuna, and R. Ramya. *A comparative analysis on linear regression and support vector regression*. in *2016 Online International Conference on Green Engineering and Technologies (IC-GET)*. 2016.

10. Chen, J., et al., *A comparison of linear regression, regularization, and machine learning algorithms to develop Europe-wide spatial models of fine particles and nitrogen dioxide.* Environment International, 2019. **130**: p. 104934.

11. Ao, Y., et al., *The linear random forest algorithm and its advantages in machine learning assisted logging regression modeling.* Journal of Petroleum Science and Engineering, 2019. **174**: p. 776–789.

12. Pal, M. and S. Parija. *Prediction of heart diseases using random forest*. IOP Publishing.

13. Hoerl, R.W., *Ridge Regression: A Historical Context.* Technometrics, 2020. **62**(4): p. 420–425.

14. Meyer, A., A. Albarghouthi, and L. D'Antoni, *Certifying robustness to programmable data bias in decision trees.* Advances in Neural Information Processing Systems, 2021. **34**: p. 26276–26288.

15. Emmert-Streib, F. and M. Dehmer, *High-Dimensional LASSO-Based Computational Regression Models: Regularization, Shrinkage, and Selection.* Machine Learning and Knowledge Extraction, 2019. **1**(1): p. 359–383.

16. Pan, S., et al., *An optimized XGBoost method for predicting reservoir porosity using petrophysical logs.* Journal of Petroleum Science and Engineering, 2022. **208**: p. 109520.

17. Wang, L., et al., *M-PINN: A mesh-based physics-informed neural network for linear elastic problems in solid mechanics.* International Journal for Numerical Methods in Engineering, 2024. **125**(9): p. e7444.

18. Qi, K. and H. Yang, *Elastic Net Nonparallel Hyperplane Support Vector Machine and Its Geometrical Rationality.* IEEE Transactions on Neural Networks and Learning Systems, 2022. **33**(12): p. 7199–7209.

19. Liu, J., et al., *Study on prediction model of liquid hold up based on random forest algorithm.* Chemical Engineering Science, 2023. **268**: p. 118383.

20. Jun, M.-J., *A comparison of a gradient boosting decision tree, random forests, and artificial neural networks to model urban land use changes: the case of the Seoul metropolitan area*. International Journal of Geographical Information Science, 2021. **35**(11): p. 2149–2167.

21. Douiba, M., et al., *An improved anomaly detection model for IoT security using decision tree and gradient boosting*. The Journal of Supercomputing, 2023. **79**(3): p. 3392–3411.

22. Louk, M.H.L. and B.A. Tama, *Dual-IDS: A bagging-based gradient boosting decision tree model for network anomaly intrusion detection system*. Expert Systems with Applications, 2023. **213**: p. 119030.

23. Seto, H., et al., *Gradient boosting decision tree becomes more reliable than logistic regression in predicting probability for diabetes with big data*. Scientific Reports, 2022. **12**(1): p. 15889.

24. Fan, J., et al., *Light Gradient Boosting Machine: An efficient soft computing model for estimating daily reference evapotranspiration with local and external meteorological data*. Agricultural Water Management, 2019. **225**: p. 105758.

25. Bentéjac, C., A. Csörgő, and G. Martínez-Muñoz, *A comparative analysis of gradient boosting algorithms*. Artificial Intelligence Review, 2021. **54**(3): p. 1937–1967.

26. Taha, A.A. and S.J. Malebary, *An Intelligent Approach to Credit Card Fraud Detection Using an Optimized Light Gradient Boosting Machine*. IEEE Access, 2020. **8**: p. 25579–25587.

27. Zulfiqar, H., et al., *Identification of cyclin protein using gradient boost decision tree algorithm*. Computational and Structural Biotechnology Journal, 2021. **19**: p. 4123–4131.

28. Saleh, E., et al., *You only design once (YODO): Gaussian Process-Batch Bayesian optimization framework for mixture design of ultra high performance concrete*. Construction and Building Materials, 2022. **330**: p. 127270.

29. Jenkins, W.F., II, P. Gerstoft, and Y. Park, *Bayesian optimization with Gaussian process surrogate model for source localization*. The Journal of the Acoustical Society of America, 2023. **154**(3): p. 1459–1470.

30. Binois, M. and N. Wycoff, *A Survey on High-dimensional Gaussian Process Modeling with Application to Bayesian Optimization*. ACM Trans. Evol. Learn. Optim., 2022. **2**(2): p. Article 8.

31. Bhati, B.S. and C. Rai. *Ensemble based approach for intrusion detection using extra tree classifier*. in *Intelligent Computing in Engineering: Select Proceedings of RICE 2019*. 2020. Springer.

32. Deng, J., et al., *An efficient extraction method of journal-article table data for data-driven applications*. Information Processing & Management, 2025. **62**(3): p. 104006.

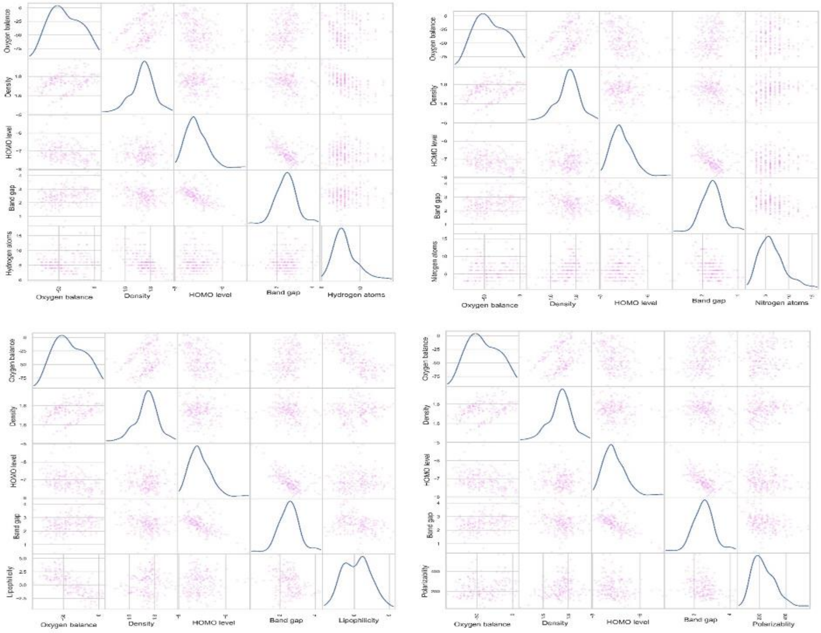

Figure S1: Scatterplot illustrating the distribution and frequency of dataset values for energetic material parameters

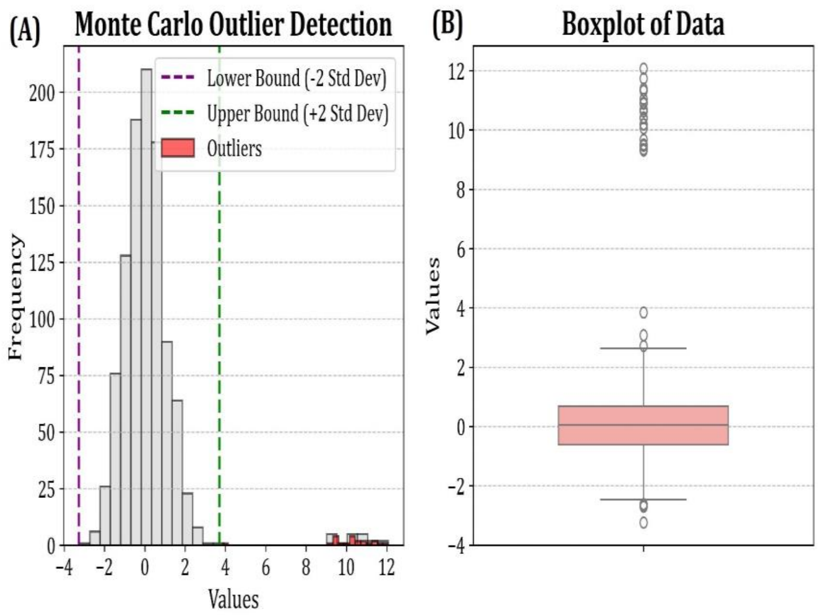

Figure S2: Visualization of dataset quality via MCOD-based outlier detection (A) and boxplot representation of output variables (B)

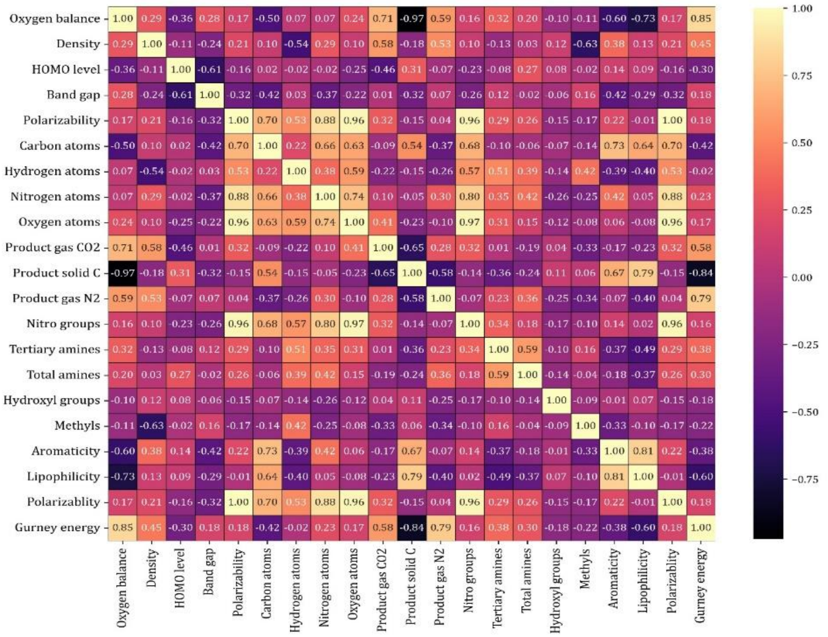

Figure S3: Pearson correlation coefficient heatmap showing pairwise relationships among all dataset parameters
